# The Interactive Stress Assessment in Basic Animal Science Training

**DOI:** 10.3390/ani11072145

**Published:** 2021-07-20

**Authors:** Theres Manthey, Stefan Nagel-Riedasch, André Dülsner

**Affiliations:** Charité—Universitätsmedizin Berlin, Corporate Member of Freie Universität Berlin and Humboldt—Universität zu Berlin, Forschungseinrichtungen für Experimentelle Medizin (FEM), Augustenburger Platz 1, 13353 Berlin, Germany; t.manthey@mailbox.org (T.M.); stefan.nagel@charite.de (S.N.-R.)

**Keywords:** stress assessment, basic animal science training, harm benefit analysis, stress in laboratory animal, animal experimentation

## Abstract

**Simple Summary:**

In basic animal science courses, an interactive stress assessment was conducted with prospective scientists with the aim of assessing the extent to which these courses lead to the acquisition of competence and expertise in recognising and assessing stress in animals. Participants significantly increased their performance in all categories. The results were also compared with those of scientists and animal caretakers. The animal caretakers achieved the best results of the three occupational groups. The results of this study illustrate, on the one hand, the high value that must be placed on the training to acquire the professional qualification and, on the other hand, how strongly the assessment of stress is influenced by subjectivity.

**Abstract:**

In order to assess the extent to which the legally prescribed training for the acquisition of animal experimentation expertise provides scientific personnel with the necessary competence and expertise to carry out a correct harm-benefit analysis in the context of animal experimentation applications, we conducted an interactive stress assessment concerning the basic animal experimentation expertise course. First, before the practical part of the course and then, after the practical part, the participants assessed images and video material of healthy and stressed animals. The results were assessed comparatively and showed a significant increase in performance in all categories (*p*-value < 0.001). In addition, the results were comparatively assessed against those of scientists already experienced in animal experiments and experienced animal caretakers in research and clinics. In all groups, the vast majority of participants were able to recognise stress in laboratory animals. A significant proportion of the participants were also able to rate the level of stress correctly according to three degrees of severity: mild, moderate and severe. Nevertheless, a small number of participants were unable to distinguish between healthy and stressed animals and thus, the stress in the individual groups was assigned very differently from the different degrees of severity. The results of this study illustrate, on the one hand, the high significance that training must have in order to acquire the expertise, and, on the other hand, how strongly the assessment of stress is influenced by subjectivity.

## 1. Introduction

Animal experiments are increasingly becoming the focus of public attention. Terms, such as transparency and alternative methods, have long been established in all areas of animal experimentation. Legal requirements and guidelines to be followed are increasing too [1]. To obtain approval for an animal experiment at all, European and national law requires that a so-called harm-benefit analysis is conducted prospectively. This means that the scientist must first consider the stress of the animals in the planned experiment and answer the question of whether the benefit that society could obtain from this experiment outweighs the stress of the animals and thus, justifies the animal experiment. From a legal point of view, this stress assessment should be objective, ethical and scientific and, if possible, based on measurable parameters [2,3]. According to Article 23 and Article 24 of the EU Directive 2010/63/EU and in Germany, Paragraph 3 of the German Animal Welfare Regulation (TierschVersV) and Section 5 of the Animal Welfare Act (TierSchG), this expertise is a prerequisite for scientific work with animals.

In order to acquire this animal experimentation expertise, scientists must complete a special training course before taking up their work in experimental animal science. In basic animal experimentation courses, according to the recommendations of the Federation of European Laboratory Animal Science Associations (Felasa), the participants learn to recognise pain, suffering and damage, to evaluate them and to react accordingly. This technical know-how is mandatory for a correct harm-benefit analysis as described above [4]. 

The aim of this study has been to investigate whether and to what extent the basic animal experimentation courses are sufficient to acquire the expertise required. On the one hand, scientists must learn to recognise stress and, on the other hand, correctly assign the stress to the three degrees of severity: mild, moderate and severe. This classification is essential in order to react correctly to stress, as it can occur in animal experiments. To answer this question, interactive stress assessments were conducted with the course participants. Subsequently, the electronically obtained data has been evaluated. In addition, the opportunity to also carry out the interactive assessment with scientists already experienced in animal experiments and professionally experienced animal caretakers in research and clinics has been seized in order to compare the results for the different occupational groups.

## 2. Materials and Methods

The interactive stress assessment took place within the framework of so-called basic animal experimentation courses of “Function A (performance of procedures on animals)” according to Article 23 of Directive 2010/63/EU. The curricula include 20 hours of theoretical and 20 hours of practical lessons, which are designed according to the Felasa guidelines and recommendations. The courses teach “7 areas: legislation, ethics and the ‘3Rs;’ the basic biology and husbandry of relevant laboratory animal species; assurance of the physiologic needs and welfare of animals without compromising scientific integrity of the investigation or procedure; animal handling techniques; conduct of basic techniques and euthanasia; recognition of lack of wellbeing and other complicating factors; anesthesia, analgesia, and basic principles of surgery; and occupational health and safety.“ The aim is for course participants to acquire a basic competence in conducting animal experiments, which is checked by the lecturers conducting the course by means of a final test [5]. Stress assessment is part of the courses. Participants must be familiar with the normal behaviour, appearance, physiological and anatomical characteristics of a species. These include feeding behaviour, body posture, grooming condition, facial expression, but also activity, vocalisation and strain variability, to name just a few examples. This knowledge is a prerequisite for recognising stress and other deviations from the norm, as animals are not able to communicate themselves. There are various assessment systems for recognising stress, but nevertheless, these systems, such as the stress assessment itself, often remain subjective [6].

Within the framework of these basic courses, we have made the stress assessment interactive. All participants were shown images and video material (*n* = 40) of healthy and distressed animals, as well as the corresponding answer options, via screen presentation and could then decide on an answer using their smartphone. The animals were mice, rats, rabbits, guinea pigs and sheep. Since different research projects also cause different forms of stress, various clinical pictures were shown, such as skin diseases, paralysis, cardiological symptoms, stereotypies and dental diseases, to name but a few.

In order to ensure that the participants were not influenced by the lecturer, the survey was carried out by only two lecturers, who defined beforehand what information the participants would receive in addition to the images and video material. Information was given that is important to recognise and correctly assess possible exposure, such as whether the animal is eating and drinking or how long the exposure has been present. The situation of the photograph was also described, such as that it was a general check of the animal cages or that the picture was taken directly after an operation.

The participants had the option to classify the animals shown as healthy or distressed and, in the case of the latter, to categorise the stress as mild, moderate or severe. In addition to the appearance of the animal, the participants also assessed behaviour, interventions and excretions, also situations such as being kept in a metabolism cage or an injection. Since animals experiencing severe stress require immediate action, it was evaluated whether healthy and suffering animals were recognised as such, as well as to what extent the participants were able to classify the stress correctly.

After the participants had given their assessment via the app, the correct answer was given by the lecturers and the case shown was discussed in detail together.

Two rounds of surveys were conducted on the group of course participants. A first survey was conducted before the practical course part (with direct work on the live animal). Afterwards, another survey took place. Here, equivalent but different images and video material were shown for assessment. The aim of the second survey was to enable assessment of whether and to what extent the course and the work with the live animal may lead to an increase in performance in the stress assessment.

The interactive stress assessment was also carried out with scientists experienced in animal experimentation (*n* = 94) and animal caretakers in research and clinics (*n* = 65), and their results were compared with those of the participants in the basic animal experimentation course (*n* = 101), to check to what extent the additional professional experience has on professional competence.

The order of the pictures was randomised. In the following, the individual questions and answers are not presented. Individual questions and their results are given as examples only. Since mild and medium levels of stress warrant more discussion as to what level of stress is certainly involved, about half of the material showed severely stressed animals (*n* = 22). None (*n* = 7), mild (*n* = 5) and medium stress (*n* = 6) were shown in about equal proportions. This ensured that the stresses shown could be safely assigned to a stress level. The stress level was based on Annex VIII of Directive 2010/63/EU and again on the Felasa recommendations, which list classic examples such as the category mild for a subcutaneous injection or severe for persistent hunchback [6].

The web-based software Kahoot! (https://kahoot.com accessed from 31 September 2019 to 3 March 2020) was used to conduct the interactive stress assessment. The results were transferred anonymously by means of aliases chosen by the participants and reported as an Excel table. The statistical analysis was carried out with the software SPSS Statistics IBM SPSS Statistics Version 25. The Bonferroni test was used to calculate significance (*p*-value < 0.05).

As with any survey, the interactive stress assessment is subject to systematic limitations. It cannot be assumed that every participant answered the questions truthfully. It is also possible that participants have previously discussed or inadvertently selected a different answer than desired. Due to the high number of participants and the chosen Bonferroni correction, it can be assumed that the limitations do not have an influence on the statistical results.

## 3. Results

### 3.1. Experience in Handling Animals 

Before the participants assessed the image and video material, they were asked whether they already had experience with animals in order to find out whether this had an influence on the correct answer rate. In the basic animal experimentation course, 64.96% of the participants stated that they already had experience with animals. Correspondingly, 35.04% stated that they had no experience. A comparison of the response rate of people with animal experience and those without showed no significant difference.

### 3.2. Influence of the Course on the Retention of Professional Competence 

The main question of the evaluation at hand was to find out to what extent participation in basic animal experimentation course could lead to obtaining the necessary competence and expertise for assessing stress in animals. A significant increase in performance was evident in the group of course participants. 

Table 1 shows how many of the questions were answered correctly before and after the course. The overall correct answer rate was 55.54%, while after the practical part of the course, 63.74% of the questions were answered correctly, which corresponds to an increase in performance of 8.2%. Healthy animals were correctly identified in 6.85% of the cases after the course and stressed animals in 10.05%. With regards to recognising that an animal is under severe stress, there was even an increase of 19.17%.

**Figure 1:**
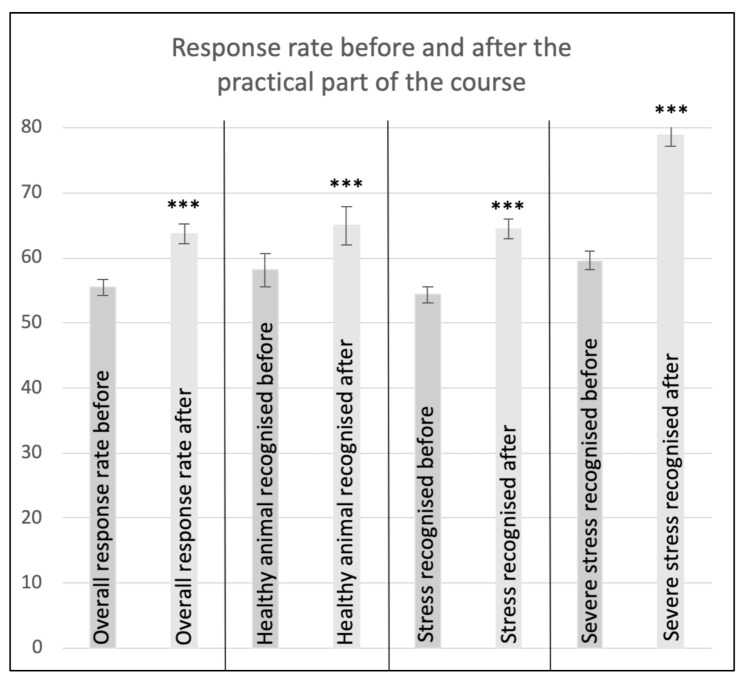
Plot of mean values and standard errors in the group of course participants before and after participation in practical teaching. Four arithmetical comparisons. *Y*-axis = mean values agreement with the answer in %. *** *p*-value < 0.001. With regards to the recognition of severely stressed animals, the highest increase in performance can be seen.

The four questions were compared with each other, and, in all cases, a significant improvement could be shown after the practical part of the course (Figure 1).

### 3.3. Comparison of the Results with Those of Scientists and Animal Keepers

**Figure 2:**
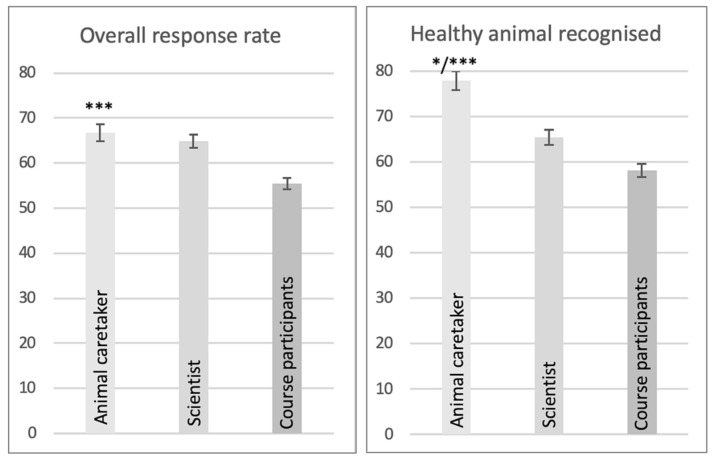

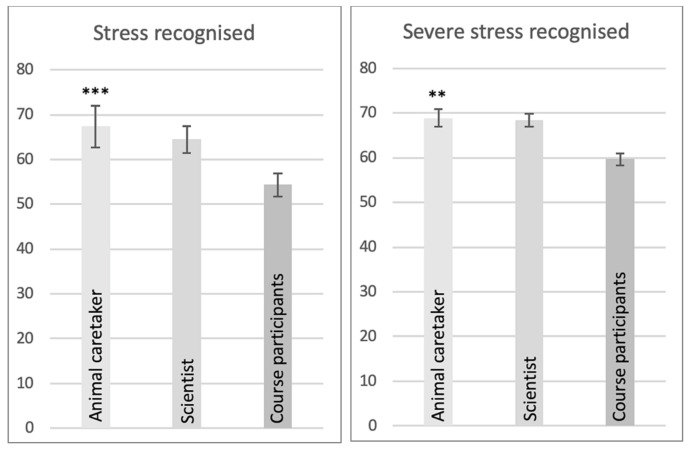
Comparison of the mean values and standard errors for the different groups. *Y*-axis = mean values agreement with the answer in %. * *p*-value < 0.05, ** *p*-value < 0.01, *** *p*-value < 0.001. The group of animal caretakers was significantly better at the individual questions, especially compared to the course participants (evaluation according to Bonferroni): general response rate *p*-value < 0.001 compared to course participants, recognition of healthy animals *p*-value 0.033 compared to scientists and *p*-value < 0.001 compared to course participants, recognition of stressed animals *p*-value < 0.001 compared to course participants and recognition of severely stressed animals *p*-value 0.001 compared to course participants.

In addition, it was evaluated whether there was a significant difference between the assessment of participants in the basic animal experimentation course and the assessment of scientists with experience in animal experimentation or animal caretakers in research and clinics, respectively (Table 2 and Figure 2).

The groups of scientists and animal keepers were also asked about their experience with animals, as it is possible that some of the participants had just started working as scientists or animal keepers. In the group of scientists working in animal experiments, 93.98% already had experience with animals, and in the group of animal caretakers this Figure reached 100%.

Table 2 shows the respective correct response rate of all three groups with regard to the four questions. Overall, the group of animal caretakers most often correctly assessed the images and video material. In this group, almost 66.74% of the questions were answered correctly. Images and video material of healthy animals were even correctly assessed in 77.99% of the cases, which is the highest correct answer rate.

The group of scientists also achieved better response rates for all four questions than the participants in the basic skills course but never achieved such good results as the animal keepers (Table 2).

The results of the three different occupational groups were compared with each other, and the animal keepers were significantly better than the course participants in assessing stress in all four questions. They were also significantly better than the scientists at recognising a healthy animal (Figure 2).

### 3.4. Subjectivity in the Assessment of Stress

In order to assess the subjectivity of the answers, the frequency with which all answer options were chosen in the individual groups was evaluated. In the groups of scientists and participants in the basic animal experimentation course, all answers of the answers possible were chosen for 51.51% of the questions. In the group of animal caretakers, on the other hand, all possible answers were chosen for only 36.36% of the questions. For example, in the case of severe stress, the wrong answers healthy or mild stress were selected less frequently or not at all. In the group of animal caretakers, there was, therefore, a significantly higher unanimity and a reduced subjectivity regarding the result (*p*-value 0.0303; Chi Quadrat Test).

## 4. Discussion 

The offer of interactive teaching materials in medical education is currently increasing immensely due to the Covid-19 pandemic. Initial studies show that their use can be useful and purposeful, but how important it is for qualitative teaching to include the lecturer for direct interaction and feedback [7,8]. Regardless of the results, it is important to mention that the interactive stress assessment also served us in an excellent way, to evaluate the knowledge level of the course participants and to identify topics that need to be trained more intensively in the practical part of the course, as also described in the above-mentioned studies. Additionally, it served excellently to increase the cooperation of the learners. In previous courses, the image and video material were answered and discussed by individual participants. Only some people actively evaluated the material, while the other part of the participants listened. In the interactive course, however, all participants assessed the animals with the smartphone-based app and, consequently, actively thought about the animals. We had the impression that this was an excellent way to increase participation that may benefit the lasting internalisation of what has been learned.

The interactive stress assessment is also used in the course to limit the use of animals to what is indispensable, to replace stressed animals completely and to improve the knowledge of the participants in the long term, which ultimately improves the welfare of the animals. In this way, stress assessment is in line with Russel and Burch’s 3Rs (Replace, Reduce, Refine) principle [9].

In the overall evaluation, a positive result was achieved in all three groups. Almost all participants, irrespective of their group membership, were able to recognise stress and, in most cases, to classify it correctly (Table 2).

The participants in the technical course were able to significantly increase their performance by up to 19.17%, which illustrates how important practical work with animals is for improving technical competence. The results of this study show that the acquisition of experimental animal science competence is necessary within the framework of basic animal experimentation courses. The increase in performance that was achieved after the practical part of the course also underlines how important it is to deal intensively with the topic of stress assessment before starting scientific work with animals.

Despite the lack of professional experience in working with animals, the participants of the basic animal science course also answered the questions correctly for the most part (Table 1: overall response rate 55.54% before the practical course part), which could possibly be attributed to a previous twenty-hour e-learning course on laboratory animal science, which also teaches fundamental aspects of stress assessment.

A possible influence of already existing experience with animals was checked; after all, almost 64.96% of the course participants stated that they already had experience with animals in the private sphere. The fact that there was no significant difference in recognising and classifying the stresses underlines the fact that private experience is not sufficient to have enough expertise in the context of animal experiment and that anyone who wants to work scientifically with animals needs training. In animal experimentation, factors such as the experimental model or the husbandry of the animals are decisive, and the persons carrying out the experiments must be able to assess the given experimental situation, habitat and the lowering of stress levels in advance, which requires adequate and, due to constant innovations, regular training of the scientific staff.

Even though it is particularly important for the laboratory animal that stress is recognised and this was the case to high proportions in almost all groups, these results must be viewed in a differentiated and cautious manner. In the group of course participants, as well as in the groups of scientists and animal keepers, it became apparent that, like the distressed, healthy animals were not recognised correctly by all participants. 

In addition to stressed animals, healthy animals should also be recognised as such, which was most frequently correctly assessed by the group of animal caretakers with a correct answer rate of 77.99%. Although the participants were informed in advance that not all the animals shown in the images and video material were distressed, some of the participants may still have had the expectation that stress was present and made their assessment accordingly. A good example of this is the picture of a healthy, pregnant rat. Here, on average, only 36% of the participants across all groups recognised the healthy animal. The remaining 64% of the participants classified the animal as being distressed. In doing so, they probably classified the visible increase in circumference as pathological and did not also assess the other relevant features, such as the shiny fur or the clear look of the animal, which tend to indicate a healthy animal.

The participants were informed in advance that mice and rats are two species that may mask mild degrees of stress so that this aspect is not always visible to the human observer. Nevertheless, approximately 30% of the course participants rated a mouse with seemingly normal behaviour, but a visible, pathologically significant girth increase, as not suffering. When asked by the lecturers, this choice was justified by the fact that the mouse did not show any deviations from normal behaviour. Another example is the image of a rat with hunchback. This bent posture is considered a definite sign of severe abdominal pain. In this case, although the majority of participants across all groups recognised severe distress (course participants 62.70%, scientists 90.12% and animal caretakers 95.56%), only the animal caretakers excluded the answers no or mild stress at all. 

In general, significantly less subjectivity and more agreement were observed in the group of animal caretakers than in the groups of scientists and course participants. Only 36.36% of the questions were all answer options selected at all. In the groups of course participants and scientists, on the other hand, all answers were selected for almost half of the questions. This result shows how strongly the assessment of pain, suffering and damage is partly subjective and emotional.

The results of the individual groups were compared with each other, and the group of animal caretakers was also most often correct in their answers compared to the other groups (Table 2). Compared to the course participants, a significantly better response rate could be shown with regard to all four questions (Figure 2). This professional group has the most frequent contact with the species assessed, which may be the cause of this very good result and may indicate competent and knowledgeable training in research and clinics.

The outcome concerning the scientists, whose correct answer rate was generally higher than that of the course participants (Table 2), can certainly be attributed to their already existing professional experience in scientific work with animals. That their response rate was lower than that of the animal caretakers may depend on the fact that a scientist usually works for years in a specific field and usually only with a specific animal species. It is conceivable, therefore, for example, that years of working on skin diseases in mice naturally makes it more difficult to correctly classify cardiological symptoms in sheep. This, in turn, would be another reason for the significantly better results of animal caretakers, whose training already covers several animal species and who usually also supervise different projects as part of their daily work. In contrast, the training in the framework of the basic animal experimentation courses, which the group of scientists had also completed before starting their work, mainly concentrates on the animal species mouse and rat, which makes it difficult to transfer this expertise to other animal species, especially if the course was a few years ago. 

Finally, it must be taken into account that probably some scientists and some animal caretakers were people with little professional experience, who were, for example, still in training or had just started their scientific work, so that their answers may have lowered the correct answer rate.

## 5. Conclusions

Even if it is certainly reassuring for the animal welfare officer that both animal caretakers and scientific staff are able to recognise stress, the so-called score sheets remain the basis for the stress assessment and the measures subsequently derived from it. A score sheet is a protocol for the examination of animals and consists of a list of trial-specific symptoms as well as the evaluation of these symptoms according to the three degrees of severity: mild, moderate, severe, and the instructions for action associated with the symptoms. This protocol is thus used for refinement [10]. The question arises, however, to what extent a score sheet can be used correctly, if the indication of stress, such as a hunchback posture, is not recognised as such by all persons working with the animal, and the assessment is often subjective, despite the rating systems. This shows, once again, that in the end, the most important criterion for adequate action in response to stress is not just a score sheet but qualified training that provides the staff with the appropriate competence. 

We consider the acquisition of expertise to be very useful for carrying out a correct harm-benefit analysis, as it is necessary for an ethical justifiability assessment since decisions on approvals for animal experiments are not made by individuals anyway. In comparison with qualified animal caretaker personnel, however, we predominantly see that professional experience and good cooperation between scientific and technical personnel are indispensable because, for the individual animal, it is not sufficient that almost all participants recognise stress, but it is of great importance that everyone who works with the animal is able to recognise pain, suffering and harm, and to react accordingly.

## Figures and Tables

**Table 1:** Comparison of the stress assessment of the course participants before and after the course for the acquisition of basic animal expertise: The course participants achieved significantly better results after the course with regards to all questions (evaluation according to Bonferroni: *p*-value < 0.001) than before the course. SD = standard deviation.

Question	Mean Value Before Course %	SD	Mean Value after Course %	SD	Performance Increase in %
Overall response rate	55.54	12.62	63.74	14.12	8.2
Healthy animal recognised	58.15	24.25	65	23.11	6.85
Stress recognised	54.35	12.66	64.40	14.47	10.05
Stress correctly classified as severe	59.64	14.82	78.81	15.26	19.17

**Table 2:** Responses to images and video material of healthy and distressed animals compared for the different groups. SD = standard deviation. The animal caretakers gave the most correct answers to all questions. The most obvious differences are in the questions recognising healthy animals and severe magnitude of stress.

Question	Group	Mean Value %	SD
Overall response rate	Animal caretaker	66.74	14.69
	Scientists	64.80	13.54
	Course participants	55.54	1262
Stress recognised	Animal caretaker	67.37	14.95
	Scientists	64.50	13.76
	Course participants	54.35	12.66
Healthy animal recognised	Animal caretaker	77.99	33.93
	Scientists	65.42	26.75
	Course participants	58.15	24.25
Stress correctly classified as severe	Animal caretaker	68.85	16.29
	Scientists	68.45	15.16
	Course participants	59.64	14.82

## Data Availability

Statement excluded, supporting data not available.
